# Detective flow imaging endoscopic ultrasound for localizing pancreatic insulinomas that are undetectable with other imaging modalities

**DOI:** 10.1055/a-2291-9116

**Published:** 2024-04-09

**Authors:** Shinichi Nihei, Yusuke Kurita, Sho Hasegawa, Kunihiro Hosono, Noritoshi Kobayashi, Kensuke Kubota, Atsushi Nakajima

**Affiliations:** 1 218758 Department of Gastroenterology and Hepatology, Yokohama City University Hospital, Yokohama, Japan; 2 218758 Department of Oncology, Yokohama City University Hospital, Yokohama, Japan

Among pancreatic neuroendocrine tumors (PNETs), pancreatic insulinomas are often particularly difficult to detect by imaging studies, making localization diagnosis difficult 1 2 . Recently, the new technique of detective flow imaging endoscopic ultrasound (DFI-EUS), which is capable of displaying minute blood flow in the entire pancreas without contrast medium, has been introduced. We report a pancreatic insulinoma that was difficult to detect with other imaging tests, and for which DFI-EUS was useful for tumor detection.

A 40-year-old woman presented with a chief complaint of dizziness. She had recurrent hypoglycemic attacks. Contrast-enhanced dynamic computed tomography, magnetic resonance imaging, fluorodeoxyglucose-positron emission tomography, and somatostatin receptor scintigraphy did not detect any lesions. EUS was performed using an ultrasound scanning system (ARIETTA 850; FUJIFILM Medical Co., Ltd., Tokyo, Japan) and convex-type endoscope (GF-UCT260; Olympus, Tokyo, Japan). B-mode EUS showed a 9-mm pale tumor with slightly higher echogenicity than the surrounding area ( Fig. 1 , Video 1 ). DFI-EUS detected a distinct multivessel tumor in the body of the pancreas ( Fig. 2 ). Contrast-enhanced EUS by Sonazoid (Daiichi-Sankyo, Tokyo, Japan) revealed a hypervascularized tumor ( Fig. 3 ). EUS-guided fine-needle aspiration was not performed because of the high risk of complications due to the main pancreatic duct interposition in the puncture line; selective arterial secretagogue injection test was positive in the body of the pancreas. The clinical diagnosis was insulinoma and a distal pancreatic body resection was performed. The final pathological diagnosis was PNET G1, insulinoma.

**Fig. 1:**
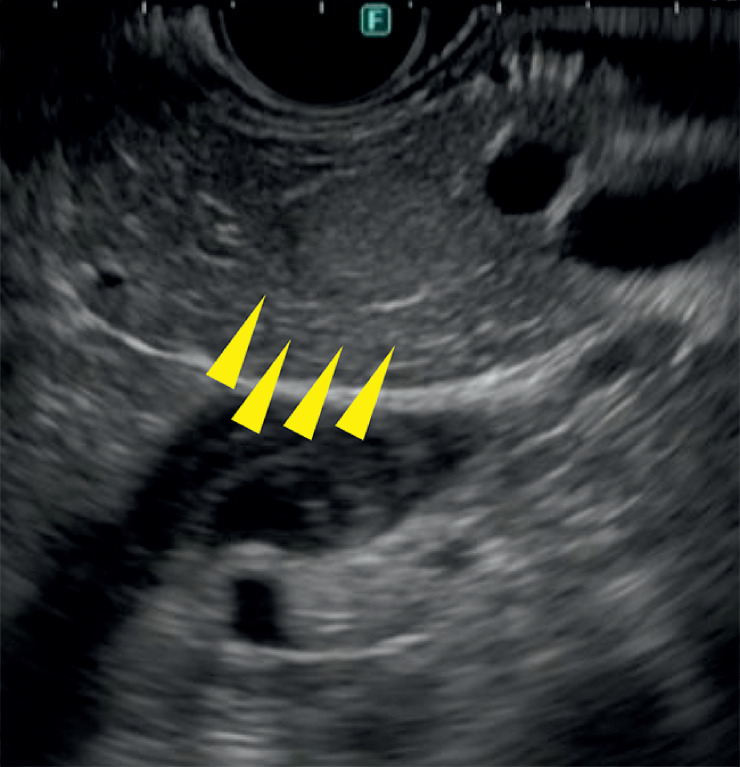
B-mode endoscopic ultrasound showed a 9-mm pale tumor (arrows) with slightly higher echogenicity than the surrounding area.

**Fig. 2:**
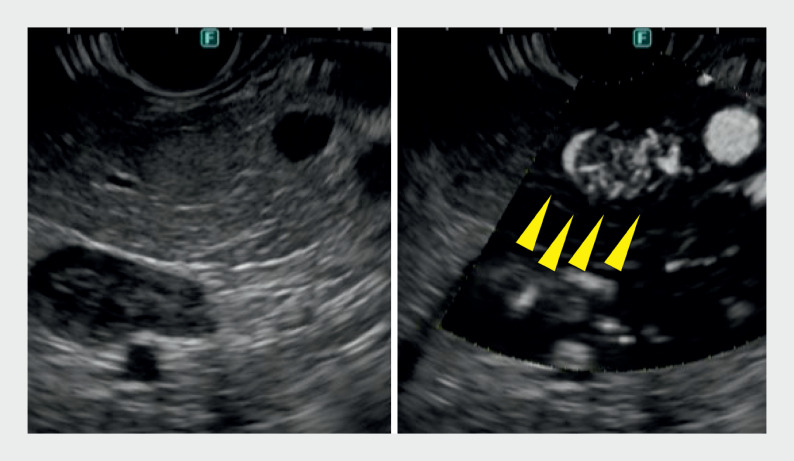
Detective flow imaging endoscopic ultrasound (DFI-EUS) detected a distinct hypervascular tumor (arrows). Strong blood flow effect was observed on the tumor surface and small vascular blood flow was observed in the interior. **a** B-mode image. **b** DFI-EUS.

**Fig. 3:**
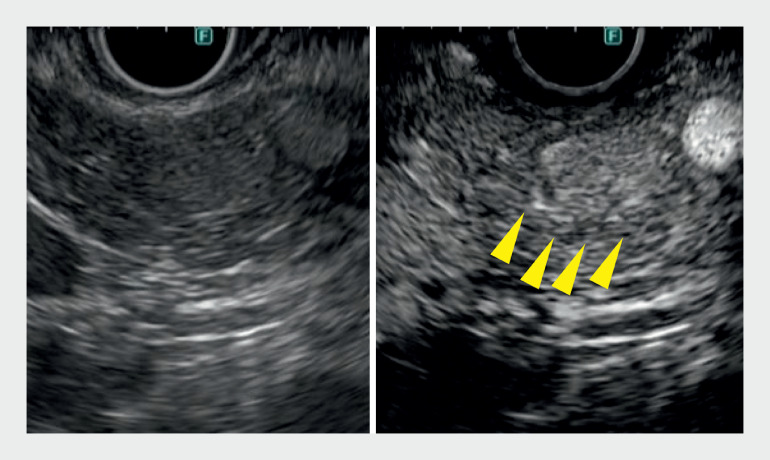
Contrast-enhanced endoscopic ultrasound depicted a mildly hypervascularized tumor (arrows), although the boundary with the surrounding area was somewhat difficult to discern. **a** B-mode image. **b** Contrast-enhanced endoscopic ultrasound.

Imaging findings of B-mode endoscopic ultrasound (EUS), detective flow imaging endoscopic ultrasound (DFI-EUS), and contrast-enhanced EUS in pancreatic insulinoma. DFI-EUS clearly delineated a tumor with more vascularity than contrast EUS.Video 1

DFI-EUS is a new method for imaging and detecting small vessels and low-velocity blood flow without the use of ultrasound contrast agents. A previous study reported the utility of DFI-EUS for pancreaticobiliary lesions 3 . In this case, DFI-EUS clearly delineated a tumor with more vascularity than contrast EUS and was useful in the diagnosis of insulinoma. DFI-EUS is useful in the evaluation of tumor blood flow in pancreatic insulinomas. While Sonazoid contrast can only adequately observe the field of view at the time of contrast injection, DFI-EUS can screen the entire pancreas. DFI-EUS may be useful for screening and localizing the entire pancreas for insulinomas and other PNETs.

Endoscopy_UCTN_Code_CCL_1AF_2AZ

## Notes

Endoscopy E-Videos https://eref.thieme.de/e-videos
*E-Videos* is an open access online section of the journal *Endoscopy* , reporting on interesting cases and new techniques in gastroenterological endoscopy. All papers include a high-quality video and are published with a Creative Commons CC-BY license. Endoscopy E-Videos qualify for HINARI discounts and waivers and eligibility is automatically checked during the submission process. We grant 100% waivers to articles whose corresponding authors are based in Group A countries and 50% waivers to those who are based in Group B countries as classified by Research4Life (see: https://www.research4life.org/access/eligibility/ ). This section has its own submission website at https://mc.manuscriptcentral.com/e-videos .
